# Effect of Overdose-Induced Hypoxia on Neurocognitive Performance in Individuals With Opioid Dependence: A Prospective Multicenter Study

**DOI:** 10.31083/AP45138

**Published:** 2026-04-08

**Authors:** Sunsha Chamakalayil, Serge Brand, Marc Vogel, Markus Gerber, Kenneth M. Dürsteler

**Affiliations:** ^1^Division of Substance Use Disorders, Psychiatric University Clinics Basel, 4002 Basel, Switzerland; ^2^Faculty of Medicine, University of Basel, 4056 Basel, Switzerland; ^3^Division of Sport and Psychosocial Health, Department of Sport, Exercise, and Health, University of Basel, 4052 Basel, Switzerland; ^4^Center for Affective, Stress and Sleep Disorders; Center for Disaster Psychiatry and Disaster Psychology, Psychiatric University Clinics Basel (UPK), 4002 Basel, Switzerland; ^5^Substance Abuse Prevention Research Center, Sleep Disorders Research Center, Kermanshah University of Medical Sciences (KUMS), 67198-51451 Kermanshah, Iran; ^6^School of Medicine, Tehran University of Medical Sciences (TUMS), 14167-53955 Tehran, Iran; ^7^Centre for Addictive Disorders, Department for Psychiatry, Psychotherapy and Psychosomatics, University Hospital of Psychiatry Zurich, 8001 Zurich, Switzerland

**Keywords:** opioid-related disorders, opioid agonist treatment, hypoxia, attention, memory, executive functions

## Abstract

**Background:**

Severe opioid dependence is associated with neurocognitive impairments, potentially aggravated by recurrent overdose-induced hypoxia and long-term opioid use–induced hypoperfusion. Although hypoxia-related impairments seem to vary with severity, duration, and frequency, findings are inconsistent. Moreover, evidence specific to opioid-related hypoxia is scarce. Given this gap, this prospective pilot study examined whether hypoxic incidents predict poorer neurocognitive performance and reduced improvement during three months of opioid agonist treatment.

**Methods:**

Forty-seven individuals with opioid dependence (mean age = 34.3 years; 27.7% female) completed a neuropsychological assessment that included 12 validated tests covering a broad range of neurocognitive domains at baseline and at study end (n = 33; 33% female).

**Results:**

The repeated-measures ANOVA yielded no significant effects of Time, Group (hypoxic incidents vs. no hypoxic incidents), or their interaction on neurocognitive performance in the domains of attention, memory, or executive functions. This suggests that changes in neurocognitive performance over time did not differ significantly between participants with and without a history of hypoxic incidents. However, post-hoc analyses indicated that hypoxic incidents requiring resuscitation may adversely affect neurocognitive functioning.

**Conclusions:**

These findings suggest that hypoxic incidents without resuscitation may not aggravate long-term neurocognitive impairments, supporting previous findings that brain cells can tolerate transient hypoxia. These results may also suggest that treatment planning should prioritize history of resuscitation incidents along with other prognostic factors such as duration of opioid use, baseline neurocognitive impairments, co-occurring mental health conditions, and socioeconomic factors. Future studies with larger samples and more precise assessments of hypoxia are essential to further clarify these findings and optimize treatment approaches.

## Main Points

1. No significant difference in attention, memory, and executive functions was found between opioid dependent individuals without and with hypoxic incidents in their history.
2. After three months of opioid agonist treatment, there was no significant improvement in attention, memory and executive functions in the same individuals.
3. Replication with larger cohorts with more precise hypoxia assessment is needed to clarify the impact of hypoxia severity on neurocognitive performance.

## 1. Introduction

Opioid dependence is a serious relapsing health condition with a well-established neurobiological basis, leading to substantial personal and public health consequences as well as ecological costs [1]. It is associated with deteriorated structural and functional neuronal changes [2, 3, 4, 5] and a wide range of impairments in neurocognitive functioning [6, 7, 8, 9]. A systematic review reported connectivity differences within brain networks involving the amygdala, anterior cingulate cortex, orbitofrontal cortex, prefrontal cortex, and nucleus accumbens in individuals with opioid dependence [10]. Furthermore, studies have demonstrated that the use of heroin and undergoing opioid agonist treatment (OAT) are commonly associated with neurocognitive impairments. These impairments persist even after prolonged periods of abstinence [7, 8, 11, 12]. However, individuals undergoing OAT perform better in several neurocognitive domains than individuals with opioid dependence who were either active opioid users or currently abstinent [13]. Specifically, impairments in attention, visuo-spatial memory, working memory, and executive functioning have been documented in these populations [6, 7, 12, 14, 15, 16, 17, 18]. Importantly, longitudinal studies on substance dependence indicate that neurocognitive improvements often emerge in the first months of treatment [19, 20, 21]. For instance, patients show improvement in verbal learning, memory, visuospatial memory, and psychomotor speed by two months of methadone treatment [21], and improvement in visual working memory by three months on buprenorphine-naloxone treatment [22].

However, the specific impact of overdose-induced hypoxic incidents on neurocognitive performance in individuals with opioid dependence is less understood. Insufficient tolerance to opioids causes overdose-related respiratory depression, and, consequently, a rapid and pronounced drop in brain oxygen levels, i.e., cerebral hypoxia [23, 24, 25], which may require resuscitation in some cases. However, the intravenous or intranasal administration of opioids can cause Cheyne-Stokes breathing, even in tolerant users. This leads to recurring transient hypoxic incidents that may be linked to brain damage [26, 27]. For example, treatment with intravenous diacetylmorphine (IV-DAM) regularly results in Cheyne-Stokes breathing patterns and mild transient hypoxic incidents [28, 29, 30]. Neurocognitive impairments resulting from these hypoxic incidents are plausible, given structural and functional alterations [27, 28, 31, 32]. More specifically, long-term opioid use-related hypoperfusion, which results in chronic hypoxia, is thought to be a factor in cell death and extensive alterations to the gray and white matter in individuals with opioid dependence [6, 33, 34, 35]. Moreover, impairments in various neurocognitive functions following opioid overdose-induced hypoxia have been reported [27, 36, 37, 38, 39, 40, 41, 42], with impairments persisting more than one year in some cases [27, 43, 44].

Numerous factors have been proposed to influence the degree of neurocognitive impairment that occurs after hypoxic incidents. Some scholars suggested that the nature and severity of the hypoxic injury were the primary factors causing the neurocognitive impairments [41, 45], while others argued that the total duration of unconsciousness following hypoxic incidents would be the primary factor for long-term neurocognitive prognosis [38]. Also, the frequency of hypoxic incidents has been identified to increase the risk for neurocognitive impairment [42, 46, 47]. Moreover, neurocognitive impairment has been linked to the site and brain areas impacted [39]. Some research, however, found no correlation between the injury cause, site of onset, or severity of injury and its prognosis over time [48]. A recent systematic review supported the hypothesis that neuropsychological outcomes after hypoxic incidents can exhibit a bimodal pattern of neurocognitive outcome, where individuals can experience significantly different levels of clinical recovery [41]. Some people may experience mild long-term neurocognitive impairment over time, whereas others may have severe impairment that can significantly interfere with everyday functioning [41, 49].

Understanding the long-term consequences of opioid-induced hypoxia on neurocognitive functioning is crucial for developing effective therapeutic approaches for opioid dependence. This study aimed to achieve this goal by comparing the neurocognitive performance of opioid-dependent individuals with a history of hypoxic incidents to that of those without such a history. We hypothesized that opioid-dependent individuals who had experienced hypoxic incidents would demonstrate significantly poorer neurocognitive performance than those with no such history. Additionally, we predicted that, after three months of comprehensive OAT, patients without a history of hypoxic incidents would show significantly greater improvements in neurocognitive performance than those with such a history.

## 2. Materials and Methods

### 2.1 Participants

Forty-seven outpatients with severe opioid dependence formed the baseline sample. Inclusion criteria were (1) age between 18 and 65 years; (2) International Classification of Diseases 10th Revision (ICD-10) diagnosis of opioid dependence as diagnosed by skilled and experienced psychiatrists and based on a thorough clinical interview [50]; (3) willingness and ability to interact and to complete forms in German, (4) willingness and ability to comply with study conditions, (5) admission to OAT with IV-DAM or oral opioids, and (6) signed written informed consent. Exclusion criteria were (1) any history of severe cerebral trauma, (2) any medical condition deemed clinically significant for the integrity of neurocognitive functioning, and (3) any present co-occurring serious mental health illness other than substance dependence, as ascertained by the thorough clinical interview assessed as mentioned above.

### 2.2 Procedures and Measures

Individuals entering OAT with IV-DAM or oral opioids at seven treatment centers located in Switzerland were approached during the period of January 2001 to December 2001 to participate in this study. While the data were collected in 2001 and have not been previously published due to a lack of statistically significant findings at the time, the study protocol and assessment methods used at the time remain consistent with current clinical standards. Given the continued relevance of IV-DAM as a treatment option and the growing emphasis on publishing clinically meaningful but nonsignificant results, the present analysis remains timely and relevant. The study’s aim and the confidential handling of sensitive data were clearly disclosed to the participants. Then, they signed the written formal informed consent. Next, individuals were tested on neurocognitive abilities both at baseline and after three months. Assessments were repeated at three months, as the cognitive improvements typically occur during the early stabilization phase [19, 21].

The neuropsychological testing was conducted in a room with a comfortable and quiet atmosphere and under consistent conditions in terms of lighting, sound, heat, and visual stimuli. The testing period was customized for each individual and was intended to minimize the impact of acute opioid administration or opioid withdrawal on test scores. Expert psychiatrists with a subspecialty in addiction medicine evaluated individuals for signs of intoxication or withdrawal prior to sessions. Participants in neuropsychological testing were not allowed to consume food, stimulating beverages, or smoke tobacco cigarettes. Participants were advised to take a 15-minute break midway through the testing sessions, which lasted approximately two hours. Following the testing sessions, the participants’ urine was collected and screened for amphetamine, cocaine, opiates, benzodiazepines, and cannabis.

Before the baseline neuropsychological examinations, the examiner interviewed the individual about their history of neurological incidents (brain injury, hypoxic unconsciousness, and resuscitation) linked to poor functional outcomes, as well as their current and past substance use. The interviews were based on a self-developed questionnaire and on a short version of the Drug and Alcohol Section of the European Addiction Severity Index [51, 52]. This instrument was also administered at the study’s end. All neuropsychological data were collected in paper-and-pencil format.

The neuropsychological battery consisted of tests with proven reliability and validity [53]. It covered the major components of neurocognitive functioning, including premorbid verbal intelligence, attention, learning and memory, cognitive flexibility, motor coordination, and information processing. The battery was designed to measure selected functional domains that are potentially impaired in extreme-altitude climbers [54, 55, 56]. Since they may experience comparable levels of hypoxia as patients with opioid dependence [57], it was employed to measure potential hypoxia-related impairments in the present study. To increase participant adherence, the entire battery was kept as short as possible, with some modifications to the standard procedures. The battery contained the following tests: Mehrfachwahl-Wortschatz-Intelligenztest ([MWT], multiple-choice vocabulary intelligence test), Complex Figure Test, Taylor Figure Test, Rey Auditory Verbal Learning Test, Rey Visual Design Learning Test, S-Word Fluency Test, Stroop Test, Five-Point Test, Goldenberg Test, d2-Test, Digit Symbol Substitution Test and Forward and Backward Digit Span of the Wechsler Memory Scale. All tests are described in detail in a recent publication [58]. In addition, sociodemographic data, including sex, age, years of heroin usage, dosage, and illness-related information, were collected from the electronic medical records.

### 2.3 Statistical Analyses

All statistical computations were performed with SPSS® 28.0 (IBM Corporation, Armonk, NY, USA) for Windows®. The level of significance was set at α < 0.05. First, neurocognitive tests were clustered into the following three domains: attention, memory, and executive functions, based on the theoretical construct of the tests and the associations between the tests [58]. Next, neurocognitive tests were aggregated to indices, i.e., an index score for attention, memory, and executive functions was built by calculating the mean for each domain using the z-scores of tests assigned to each domain. Then, separate repeated-measures analyses of variance (rANOVAs) were calculated to evaluate the effect of hypoxic incidents on neurocognitive performance over time for the domains of attention, memory, and executive functions. Four cases at baseline and two additional cases at study end with missing values on hypoxic incidents were removed from the analyses. The within-subject factor was Time (baseline and study end) and the between-subject factor was Group (no hypoxic incidents vs. one or more hypoxic incidents). Dependent variables included performance in the domains of attention, memory, and executive functions. All assumptions for rANOVAs were tested. Normality was assessed with Shapiro-Wilk tests and Q-Q plots for each variable and group, and homogeneity of variances was evaluated with Levene’s tests. The test of sphericity was not applicable because the within-subject factor time had only two levels. The assumption of sphericity is inherently satisfied in this case, and no corrections to degrees of freedom were required. An intent-to-treat approach was applied. Fourteen participants could not be retrieved for the follow-up assessments for reasons of treatment dropout, for personal reasons, and for reasons of current episode of major depression. T-tests showed no systematic and biased results in favor of dropouts or those still in the study. Missing follow-up values were dealt with using the last observation carried forward (LOCF) method to maintain a balanced dataset in this pilot sample. Since improvement over time was anticipated, LOCF was selected as a conservative approach likely to attenuate estimated effects.

To identify homogenous subgroups based on neurocognitive performance in the domains of attention, memory, and executive functions and to determine if the severity of hypoxic incidents influenced neurocognitive performance, post-hoc exploratory analyses in the form of K-means clustering (Euclidean distance) were conducted. To this end, K-means analysis clustered participants based on their neurocognitive performance in the three domains and investigated their relationship with the presence or absence of prior hypoxic incidents and resuscitation incidents, where hypoxia tends to be more severe. Data was screened for outliers through visual inspection (scatterplots, boxplots, and histograms) and extreme value analysis. One outlier was identified over all three performance variables (attention, memory, and executive functions) and subsequently removed from the analyses. The ideal number of clusters was determined based on interpretability and inter-cluster distances. ANOVA was used to validate differences in neurocognitive performance between clusters, and chi-square tests assessed the relationship between cluster membership and the presence or absence of hypoxic or resuscitation incidents.

## 3. Results

### 3.1 General Information on the Sample

Table 1 gives a descriptive statistical overview of sociodemographic and illness-related information for baseline and study end as well as stratified by the group variable (no hypoxic incidents vs. hypoxic incidents).

**Table 1.** **Descriptive statistical overview of sociodemographic and illness-related information**.

	Timeline					
	Baseline			Study end		
	Total	No hypoxic incidents	Hypoxic incidents	Total	No hypoxic incidents	Hypoxic incidents
N; (n females)	43 (13)	24 (7)	19 (4)	31 (9)	18 (6)	13 (3)
	Mean (SD)					
Dose of opioid (mg)	111.25 (71.61)	83.75 (5.30)	97.50 (32.33)	126.90 (79.46)	123.04 (86.98)	123.33 (43.80)
BDI; depression; self-rating	13.74 (9.33)	12.25 (9.26)	15.42 (9.82)	12.94 (9.18)	10.67 (8.25)	16.75 (10.31)
GSI-score of SCL-90-R; psychiatric symptoms; self-rating	60.76 (12.88)	60.40 (13.15)	61.18 (12.94)	60.48 (13.64)	59.19 (14.45)	62.36 (12.83)
Age in years	34.04 (7.30)	34.17 (6.44)	32.37 (6.04)	34.24 (8.04)	34.36 (7.09)	32.21 (6.24)
Heroin use in years	10.83 (5.18)	10.52 (5.13)	11.50 (2.13)	10.61 (5.32)	9.81 (5.50)	11.96 (5.41)

Note. SD, standard deviation; Dose, methadone equivalent dose in mg using conversion ratio of 1:4 for injectable diacetylmorphine and 1:8 for oral diacetylmorphine; BDI, Beck’s Depression Inventory; GSI, Global Severity Index; SCL-90-R, Symptom Checklist-90-Revised.

Table 2 shows the mean z-scores (standard deviations) for performance on neurocognitive tests in three domains: attention, memory, and executive functions at baseline and at the end of the study, for participants without hypoxic incidents and those with hypoxic incidents.

**Table 2.** **Descriptive statistics of neurocognitive performance scores at baseline and study end**.

Domain	Group	Baseline mean (SD)	Study end mean (SD)	Cohen’s d
Attention	No hypoxic incidents (n = 24)	0.22 (1.02)	0.24 (1.06)	0.52
	Hypoxic incidents (n = 19)	–0.30 (0.99)	–0.22 (0.80)	0.48
Memory	No hypoxic incidents (n = 24)	0.11 (1.07)	0.12 (1.20)	0.32
	Hypoxic incidents (n = 19)	–0.22 (0.96)	–0.20 (0.76)	0.31
Executive functions	No hypoxic incidents (n = 24)	0.17 (1.08)	0.12 (1.07)	0.40
	Hypoxic incidents (n = 19)	–0.24 (0.95)	–0.03 (0.87)	0.15

Three separate rANOVAs were conducted to evaluate the effects of hypoxic incidents on neurocognitive performance over time. To account for differences in intelligence as a confounding factor, estimated premorbid intelligence (MWT) scores were compared between groups (no hypoxic incidents: M = 29.26 (standard deviation [SD] = 3.25); hypoxic incidents: M = 28.53 (SD = 3.12)). An independent samples *t*-test yielded no significant difference, t(40) = 0.743, *p* = 0.46, *d* = 0.23.

Table 3 displays the results of the rANOVAs for the effect of Time (baseline, study end), Group (no hypoxic incidents, hypoxic incidents), and of Time × Group interaction on the domains of neurocognitive performance (attention, memory, executive functions).

**Table 3.** **Results for ANOVAs for the effects of TIME (baseline, study end), GROUP (no hypoxic incidents, hypoxic incidents) and TIME × GROUP interaction on the domains of neurocognitive performance (attention, memory, executive functions)**.

Attention	*F*	*df*	*p*-value	*ηp* ^2^
Time	0.36	(1, 41)	0.55	0.01
Group	2.85	(1, 41)	0.10	0.07
Time × Group	0.18	(1, 41)	0.67	0.00
Memory	*F*	*df*	*p*-value	*ηp* ^2^
Time	0.03	(1, 41)	0.88	0.00
Group	1.13	(1, 41)	0.29	0.03
Time × Group	0.00	(1, 41)	0.99	0.00
Executive functions	*F*	*df*	*p*-value	*ηp* ^2^
Time	1.14	(1, 41)	0.29	0.03
Group	0.89	(1, 41)	0.35	0.02
Time × Group	2.80	(1, 41)	0.10	0.06

#### 3.1.1 Attention

The main effect of Time was not significant, *F*(1,41) = 0.36, *p* = 0.55, η*p*^2^ = 0.01, indicating no overall change in attention scores over time. The main effect of Group was not significant, *F*(1,41) = 2.85, *p* = 0.10, η*p*^2^ = 0.07, indicating no difference between the two groups. The Time × Group interaction was not significant, *F*(1,41) = 0.18, *p* = 0.67, η*p*^2^ = 0.00, suggesting that the change in attention scores did neither change over time, nor within and between the two groups. The no hypoxic incidents group showed a minor descriptive, but not statistically significant, improvement (M = 0.22 to M = 0.24); the hypoxic incidents group also showed a minor descriptive, but not statistically significant, improvement (M = –0.30 to M = –0.22).

#### 3.1.2 Memory

The main effect of Time was not significant, *F*(1,41) = 0.03, *p* = 0.88, η*p*^2^ = 0.00, indicating no overall change in memory scores over time. The main effect of Group was not significant, *F*(1,41) = 1.13, *p* = 0.29, η*p*^2^ = 0.03, indicating no difference between the two groups. The Time × Group interaction was not statistically significant, *F*(1,41) = 0.00, *p* = 0.99, η*p*^2^ = 0.00, indicating that changes in memory scores did neither statistically significantly change over time, nor within and between the two groups. The no hypoxic incidents group showed a minor descriptive, but not statistically significant, improvement (M = 0.11 to M = 0.12), while the hypoxic incidents group showed a similar minor improvement (M = –0.22 to M = –0.20).

#### 3.1.3 Executive Functions

The main effect of Time was not statistically significant, *F*(1,41) = 1.14, *p* = 0.29, η*p*^2^ = 0.03, indicating no overall change in executive functions scores over time. The main effect of Group was not significant, *F*(1,41) = 0.89, *p* = 0.35, η*p*^2^ = 0.02, indicating no difference between the two groups. The Time × Group interaction was not statistically significant, *F*(1,41) = 2.80, *p* = 0.10, η*p*^2^ = 0.06. Although the result did not meet the threshold for statistical significance, an observed medium effect size suggests a moderate interaction effect. This indicates that the change in executive functioning scores over time may differ between the two groups. The no hypoxic incidents group showed a minor descriptive, but not statistically significant decrease (M = 0.17 to M = 0.12), whereas the hypoxic incidents group showed a more notable descriptive, but not statistically significant improvement (M = –0.24 to M = –0.03).

### 3.2 Post-hoc Analyses

Table 4 shows the mean z-scores (standard deviations) for performance on neurocognitive tests in the three domains: attention, memory, and executive functions, at baseline and at the end of the study, for participants without resuscitation incidents and those with resuscitation incidents in their history. To account for differences in intelligence as a confounding factor, estimated premorbid intelligence (MWT) scores were compared between groups (no resuscitation incidents: M = 29.26 (SD = 3.16); resuscitation incidents: M = 28.00 (SD = 3.16)). An independent samples *t*-test revealed no significant difference, *t*(40) = 1.13, *p* = 0.26, *d* = 0.40.

**Table 4.** **Descriptive statistics of neurocognitive performance scores at baseline and study end**.

Domains	Group	Baseline mean (SD)	Study end mean (SD)	Cohen’s d
Attention	No resuscitation incidents (n = 32)	0.26 (0.94)	0.20 (1.01)	1.11
	Resuscitation incidents (n = 11)	–0.78 (0.91)	–0.46 (0.62)	0.71
Memory	No resuscitation incidents (n = 32)	0.02 (1.04)	0.09 (1.10)	0.19
	Resuscitation incidents (n = 11)	–0.18 (0.99)	–0.34 (0.75)	0.42
Executive Functions	No resuscitation incidents (n = 32)	0.11 (1.04)	0.14 (1.00)	0.47
	Resuscitation incidents (n = 11)	–0.37 (0.96)	–0.21 (0.92)	0.36

The K-means cluster analysis identified three distinct clusters for the domains of attention, memory, and executive functions. Table 5 presents the final cluster centers. Cluster 1 displayed higher neurocognitive functioning, cluster 2 showed moderate neurocognitive impairments (particularly in the domain of memory), and cluster 3 had substantial impairments in attention and executive functions. Clusters 1 and 3 exhibited the largest distance (d_13_ = 3.32), indicating the greatest neurocognitive separation; clusters 2 and 3 showed moderate separation (d_23_ = 2.19), whereas clusters 1 and 2 were closest (d_12_ = 1.70). ANOVAs among clusters were significant across all domains: attention: *F*(2,43) = 39.34, *p* < 0.001, η*p*^2^ = 0.65; memory: *F*(2,43) = 25.5, *p* < 0.001, η*p*^2^ = 0.52; executive functions: *F*(2,43) = 22.97, *p* < 0.001, η*p*^2^ = 0.52.

**Table 5.** **Final cluster centers (Z-Scores)**.

Domains	Cluster 1	Cluster 2	Cluster 3
	(n = 26)	(n = 14)	(n = 6)
Attention	0.40	–0.30	–1.84
Memory	0.50	–0.88	–0.52
Executive functions	0.41	–0.30	–1.82

Chi-square analysis showed no significant relationship between hypoxic incidents and neurocognitive clusters (χ^2^ (2, 42) = 2.26, *p* = 0.32). However, chi-square analysis revealed a statistically significant association between resuscitation incidents and neurocognitive clusters (χ^2^ (2, 42) = 8.58, *p* = 0.01). Participants who had resuscitation incidents were disproportionately overrepresented in the lowest neurocognitive functioning cluster (Cluster 3), indicating that hypoxic incidents with following resuscitation could be associated with more severe neurocognitive impairment. Table 6 shows the cluster membership by hypoxic and resuscitation incidents.

**Table 6.** **Cluster membership by hypoxic incidents and resuscitation incidents**.

Cluster	No hypoxic incidents	Hypoxic incidents	No resuscitation incidents	Resuscitation incidents	Total
Cluster 1	15 (65%)	8 (42%)	19 (61%)	4 (36%)	23 (55%)
Cluster 2	6 (26%)	8 (42%)	11 (35%)	3 (27%)	14 (33%)
Cluster 3	2 (9%)	3 (16%)	1 (3%)	4 (36%)	5 (12%)
Total	23	19	31	11	42

## 4. Discussion

This study investigated whether opioid-dependent individuals without a history of hypoxic incidents performed better in neurocognitive functioning than those with a history of hypoxic incidents. The study also examined whether opioid-dependent individuals without a history of hypoxic incidents improved more following a three-month comprehensive OAT program than those with a history of hypoxic incidents.

Our first hypothesis was that opioid-dependent individuals without a history of hypoxic incidents would perform better than those who had experienced hypoxic incidents. We did not confirm this hypothesis. As shown in Table 3, there was no significant difference between the groups in any of the three domains (attention, memory, and executive functions), for which trivial to medium effect sizes were found. Therefore, the current findings do not corroborate the hypothesis that hypoxic incidents profoundly affect attention, memory, and executive functioning in the long term in opioid-dependent individuals. However, our post-hoc analysis shows that individuals with a history of resuscitation incidents are disproportionately overrepresented in the cluster with the lowest neurocognitive performance, suggesting that the resuscitation incidents may have an impact on neurocognitive performance. This finding is based on a small subgroup and should be interpreted with caution. Nevertheless, we believe these results add to the findings of preclinical studies, showing that brain cells can tolerate robust, but transient hypoxia for short periods of time [24, 59, 60]. This may not be the case for severe levels of hypoxia during resuscitation incidents. Based on current understanding, hypoxia requiring resuscitation may injure the brain through glutamate-mediated excitotoxicity, oxidative stress, and mitochondrial dysfunction, with high vulnerability of the hippocampus and associated cortices [61, 62, 63]. After global hypoxia, white-matter microstructural injury, which relates to worse neurocognitive functioning, can be detected [64]. Disruptions of glutamate-gamma-aminobutyric acid (GABA) signaling and synaptic functioning after global hypoxia may further impair network function and underlie these neurocognitive effects [65].

According to our second hypothesis, opioid-dependent individuals without a history of hypoxic incidents would improve more in neurocognitive functions following a three-month comprehensive treatment program than opioid-dependent individuals with a history of hypoxia. Our findings refuted this hypothesis: There was no statistically significant difference between the groups over time, as Table 3 illustrates, and trivial to medium effect sizes were found for each of the three domains—attention, memory, and executive functions. Therefore, the present data do not support the hypothesis that hypoxic incidents in a patient’s history would affect the improvement of neurocognitive functioning in attention, memory, and executive functioning. However, in the domain of executive functioning a moderate effect size was observed in Group × Time interactions. These findings may suggest a possible influence of hypoxic incidents on neurocognitive performance over time. The difference may not have reached statistical significance because of the small sample size and the resulting low statistical power. Therefore, although the current findings do not permit definitive conclusions, they do not eliminate the possibility that hypoxic incidents could have long-term effects on executive functioning in opioid-dependent individuals.

We believe that the current findings are significant for several reasons: First, these findings imply that in general treatment programs for opioid dependence may not need to be differentiated based on the presence or absence of hypoxic incidents, because the lack of significant differences in neurocognitive performance between opioid-dependent individuals with and without hypoxic incidents may challenge the theory that hypoxic incidents exacerbate neurocognitive impairments. However, the current findings may suggest that treatment planning for patients with a history of resuscitation should account for potential neurocognitive impairment. Second, this suggests that clinicians may focus on thorough evaluations that consider a wider range of variables impacting neurocognitive functioning. These factors may include the duration and intensity of opioid use, co-occurring mental health disorders, socioeconomic factors, and more. Clinicians can also distribute resources more evenly. Third, these results highlight the importance of interventions tailored to specific individuals and their identified risk factors or impairments. Fourth, the inconsistencies with some previous research might be due to differences in the severity of hypoxic incidents and composition of the sample, methods of assessing hypoxia and resuscitation, neurocognitive tests administered, and follow-up timing [6, 27, 66, 67, 68]. Therefore, replication studies are needed to confirm these results and support the significance of hypoxic incidents (or lack thereof) in neurocognitive performance among opioid-dependent individuals. Lastly, the detection of trivial to medium effect sizes, even though not significant, suggests the need for larger sample sizes or different statistical methods in future studies to detect subtle differences, particularly on the impact of hypoxia severity on neurocognitive functioning. Also, this encourages longitudinal studies to track cognitive changes over time and understand the long-term impacts of opioid dependence and hypoxic incidents.

Despite the importance of the present findings, several limitations should be considered. First, the sample size may have been insufficient to identify subtle but significant differences in neurocognitive functioning between individuals who experienced hypoxic incidents and those who did not, particularly the difference in executive functioning over time. Also, our finding that individuals with a history of resuscitation were overrepresented in the lowest neurocognitive functioning cluster is based on a very limited sample size. Moreover, although our analysis identified three distinct clusters, we acknowledge that cluster structure is method-dependent. Alternative cluster approaches and validity criteria, and different distance metrics could yield different partitions. Our results should therefore be interpreted with appropriate caution. If there are significant effects, it may be challenging to detect them due to low statistical power resulting from the small sample size. Further research with larger samples is thus needed to explore our findings more conclusively. Also, the LOCF-method, used to handle missing follow-up data, assumes stability after dropout and can bias estimates when missingness is informative. Findings should therefore be interpreted as conservative with respect to time and interaction effects. Future confirmatory studies should adopt more robust handling of missing data. Second, the heterogeneity of the sample could have introduced variability that masked the effects of hypoxic incidents. Variations in the severity of opioid dependence, duration of use, types of opioids used, and the presence of co-occurring conditions, such as mental health disorders (e.g., attention-deficit/hyperactivity disorder [ADHD], depressive disorders, personality disorders), could have influenced the results. Third, since we did not systematically assess the level or duration of hypoxic incidents, we may not have adequately differentiated between the hypoxic incidents, which could have varying impacts on neurocognitive outcomes [60]. A recent systematic review found that moderate hypoxia exposure may have positive effects on measures of neurocognitive and neurological functioning [60]. Future studies are recommended to use objective and systematic measures of hypoxia. Fourth, uncontrolled confounding variables, such as emotional regulation, impulsivity, and co-occurring mental health disorders (e.g., ADHD, personality disorders), were not thoroughly assessed and might have biased the results. Fifth, baseline neurocognitive function before the onset of opioid use and hypoxic incidents was not measured or controlled for, potentially confounding differences attributable to hypoxia with pre-existing cognitive differences. Addressing these limitations in future research could help clarify the relationship between hypoxic incidents and neurocognitive performance in opioid-dependent individuals and explain discrepancies with prior findings.

## 5. Conclusions

The results of this study may contribute to a more comprehensive understanding of the neuropsychological profile of individuals with opioid dependence, particularly regarding the potential impacts of hypoxic incidents. Additionally, it may provide new insights into how hypoxic incidents affect neurocognitive functioning in this population. We claim that the present findings are of importance because they can contribute to the design of specific neurocognitive treatment options for opioid-dependent patients. If hypoxic incidents do not significantly impact neurocognitive outcomes, these results could imply that treatment programs could be administered more broadly and focus on other contributing factors. Clinically, routine screening for hypoxia or resuscitation-related risk and neurocognitive impairments, coupled with simple accommodations (clear written summaries, reminders, simplified plans), may support treatment engagement and improve outcomes. This approach can improve the overall quality of life for opioid-dependent patients by assisting them in managing their neurocognitive impairments in day-to-day living.

## Data Availability

The data presented in this study are available on reasonable request to experts in the field. Such a request consists, among others, of specific hypotheses and the proof of secure and protected data handling. The corresponding team decides whether to accept or decline a request.

## Abbreviations

OAT, opioid agonist treatment; IV-DAM, intravenous diacetylmorphine; LOCF, last observation carried forward; SD, standard deviation; BDI, Beck’s Depression Inventory; GSI, Global Severity Index; SCL-90-R, Symptom Checklist-90-Revised; MWT, Mehrfachwahl-Wortschatz-Intelligenztest; GABA, glutamate-gamma-aminobutyric acid; ADHD, attention-deficit/hyperactivity disorder.
